# 3-O-Ethyl Ascorbic Acid and Cannabigerol in Modulating the Phospholipid Metabolism of Keratinocytes

**DOI:** 10.3390/antiox13111285

**Published:** 2024-10-24

**Authors:** Iwona Jarocka-Karpowicz, Izabela Dobrzyńska, Anna Stasiewicz, Elżbieta Skrzydlewska

**Affiliations:** 1 Department of Analytical Chemistry, Medical University of Białystok, Mickiewicza 2D, 15-222 Białystok, Poland; iwona.jarocka-karpowicz@umb.edu.pl (I.J.-K.); anna.stasiewicz@umb.edu.pl (A.S.); 2 Laboratory of Bioanalysis, Faculty of Chemistry, University in Białystok, Ciołkowskiego 1K, 15-245 Białystok, Poland; izadob@uwb.edu.pl

**Keywords:** keratinocytes, UVB radiation, 3-O-ethyl ascorbic acid, cannabigerol, eicosanoids, endocannabinoids, lipid peroxidation

## Abstract

Phospholipids and their metabolites play an important role in maintaining the membrane integrity and the metabolic functions of keratinocytes under physiological conditions and in the regeneration process after exposure to high-energy UVB radiation. Therefore, in the search for compounds with a protective and regenerative effect on keratinocyte phospholipids, the effectiveness of two antioxidant compounds has been tested: a stable derivative of ascorbic acid, 3-O-ethyl ascorbic acid (EAA) and cannabigerol (CBG), both of which are primarily located in the membrane structures of keratinocytes. In addition, this study has demonstrated that EAA and CBG, especially in a two-component combination, enhance the antioxidant properties of keratinocytes and reduce lipid peroxidation assessed at the level of MDA (malondialdehyde)/neuroprostanes. Moreover, by reducing the activity of enzymes that metabolise phospholipids, free PUFAs (polyunsaturated fatty acids) and endocannabinoids (PLA2; phospholipase A2, COX1/2; cyclooxygenases 1/2, LOX-5; lipoxygenase 5, FAAH; fatty acid amide hydrolase, MAGL; monoacylglycerol lipase), antioxidants have been found to regulate the levels of endocannabinoids (AEA; anandamide, 2-AG; 2-arachidonoylglycerol, PEA; palmitoylethanolamide) and eicosanoids (PGD2; prostaglandin D2, PGE2; prostaglandin E2, 15-d-PGJ2; 15-deoxy-Δ12,14-prostaglandin J2, 15-HETE; 15-hydroxyeicosatetraenoic acid), that are enhanced by UVB radiation. The metabolic effect of both groups of PUFA metabolites is mainly related to the activation of G protein-related receptors (CB1/2; cannabinoid receptor 1 and 2, PPARγ; peroxisome proliferator-activated receptor gamma, TRPV1; transient receptor potential cation channel subfamily V member 1), the expression of which is reduced under the influence of EAA, CBG, and especially the two-component combination. It promotes the regeneration of keratinocyte metabolism disrupted by UVB, particularly in relation to redox balance and inflammation.

## 1. Introduction

The skin is a mechanical barrier that separates the human body from the external environment and is therefore the first line of defence against exogenous factors, such as pathogens or UV radiation [1]. The outermost layer of the skin is the epidermis, where keratinocytes predominate [2]. Due to intercellular connections, those cells prevent the penetration of melanin into the deeper layers of the skin, thus protecting them against UV radiation [3], including carcinogenic UVB radiation [4,5].

The electromagnetic energy of UVB radiation increases the generation of ROS [6], which results in structural and functional modifications of the main components of cells, including membrane proteins and phospholipids [7]. Phospholipids, as the main structural elements of keratinocyte membranes, play a crucial role in maintaining the metabolic functions of those cells [8]. It has inter alia been shown that phosphatidylcholines are involved, among others, in cell proliferation and differentiation [8], while phosphatidylserines and phosphatidylethanolamines participate in membrane fusion and cell apoptosis [9]. They are responsible for maintaining the natural pH of the skin, and by forming a protective film on its surface, help prevent water loss and contribute to the skin’s flexibility [10].

However, under the influence of enzymes (PLA2, COX1/2, LOXs, FAAH, MAGL), the activity of which increases due to UVB radiation, phospholipids undergo enhanced metabolism, leading to the generation of lipid mediators such as eicosanoids and endocannabinoids [11]. Those mediators play a role in regulating ROS levels, inflammation, and the body’s immune response [12]. Moreover, both phospholipids and free polyunsaturated fatty acids (PUFAs) undergo peroxidation in ROS-dependent reactions, generating products of oxidative cyclisation, including neuroprostanes, isoprostanes, and reactive aldehydes such as MDA and 4-HNE [13]. By forming adducts with proteins, lipids, and DNA, those products contribute to cellular metabolism disorders and the development of skin diseases, including psoriasis, atopic dermatitis [14], and even cancer [15]. In consequence, there is an ongoing search for compounds/substances that, without generating side effects, could be used for the purpose of protection and/or regeneration of the metabolism of skin cells disrupted by the exposure to UVB radiation [16].

Ascorbic acid, necessary for the proper synthesis of collagen and the differentiation of keratinocytes, as well as the protection of keratinocytes against the metabolic effects of overproduction of ROS, is one of the cellular antioxidants and ingredients often used in skin preparations [17]. However, its physicochemical properties, including its hydrophilic nature, do not favour its penetration through the lipid bilayer into the cells, which reduces its effectiveness [18]. Its derivative seems to be more effective, namely 3-O-ethyl ascorbic acid (EAA), characterised by a metabolic efficiency similar to vitamin C, that, thanks to its greater lipophilicity, penetrates the lipid barrier more effectively [19,20]. EAA, used together with lactic acid, has been shown to reduce the level of histone γ-H2AX that is a marker of DNA damage induced by UVB radiation [20]. However, EAA may cause allergic contact dermatitis [21,22], therefore, its use in combination with a compound that has antioxidant and anti-inflammatory properties, such as the phytocannabinoid—cannabigerol (CBG), may generate beneficial effects. However, it is known that CBG has antioxidant and anti-inflammatory effects also in response to UV radiation [23,24,25] and prevents allergic skin reactions [26]. Moreover, CBG effectively limits transport through keratinocyte membranes, thereby reducing the distribution of protoporphyrin IX in the skin and thus preventing phototoxicity associated with erythropoietic protoporphyria [27]. However, being a partial agonist of the PPARγ receptor, CBG indirectly protects membrane lipids against damage by preventing IκB-α phosphorylation and translocation of nuclear factor-κB (NF-κB) as well as regulating the MAP kinase pathway [28]. Moreover, the intensification of collagen and elastin biosynthesis strengthens the functions of the skin barrier [24].

Therefore, taking into account the importance of the proper lipid structure of basic skin cells for their physiological functioning, as well as counteracting the development of oxidative stress and inflammation as a result of UVB radiation to which humans are constantly exposed, the aim of this study has been to assess the protective and regenerative effect of 3-O-ethyl ascorbic acid (EAA) and cannabigerol (CBG), both as single compounds and in a two-component combination, on the phospholipid metabolism of keratinocytes exposed to UVB. The obtained results may indicate new strategies for protecting the skin against solar radiation and become a promising starting point for further research.

## 2. Materials and Methods

### 2.1. Materials

#### 2.1.1. Cell Cultures

Human keratinocytes (CDD 1102 KERTr—CRL-2310) used for the experiment purpose were purchased from the American Type Culture Collection (ATCC, Manassas, VA, USA). Keratinocytes were cultured by means of a specialised medium for keratinocyte (Serum-Free Medium), enriched with Bovine Pituitary Extract (10%), human recombinant Epidermal Growth Factor (5 µg/L), and antibiotics (penicillin and streptomycin). Antibiotics were added to the medium to protect the cells from bacterial contamination. Keratinocyte culture was continued until the keratinocytes (passage 53) reached 70% confluence.

#### 2.1.2. UVB Radiation and Treatment

Keratinocytes were irradiated with UVB radiation (312 nm) by means of six lamps with a power of 6 W (Bio-Link Crosslinker BLX 312; Vilber Lourmat, Eberhardzell, Germany), which corresponds to 60 mJ/cm^2^. The UVB radiation power was selected based on the performed survival test (for 75 + 5% cells viability) [29]. The control cells were incubated in parallel without irradiation. After irradiation, cells were immediately incubated for 24 h under standard conditions.

In order to assess the effect of 3-O-ethyl ascorbic acid (EAA) and cannabigerol (CBG) on keratinocytes under the control condition and/or after the exposure to UVB radiation, the tested cells were incubated for 24 h in the medium supplemented with EAA (150 µM; TCI, Tokyo, Japan) and/or CBG (1 µM; Biokonopia SA, Nidau, Switzerland). The solutions of the tested compounds were prepared in ethanol, the final concentration of which in the medium was 0.3%. The ethanol solution of the same concentration, but without the tested compounds, was added to the cells of the control group to eliminate its possible effect on the cells. The concentrations of the tested compounds were selected based on the survival test (MTT assay [29]) results, both for the group without radiation where keratinocytes were treated only with EAA or/and CBG and the UVB group where EAA or/and CBG was added immediately after the exposure of keratinocytes to UVB radiation (Figure S1 from Supplementary File). EAA or CBG in selected concentrations did not reduce cell viability (Figure S2 from Supplementary File).

The keratinocytes in the experiment were divided into eight groups that were created as follows:

- Keratinocytes not exposed to UVB radiation
- Control: cells incubated in the standard medium (for 24 h);
- EAA: cells incubated in the medium enriched with 150 µM of EAA (for 24 h);
- CBG: cells incubated in the medium enriched with 1 µM of CBG (for 24 h);
- EAA + CBG: cells incubated in the medium enriched with 150 µM of EAA and 1 µM of CBG (for 24 h).

The keratinocytes exposed to UVB radiation [60 mJ/cm^2^]

- UVB: cultured for 24 h after the exposure in the standard medium (with 0.3% ethanol);
- UVB + EAA: immediately after UVB radiation the cells were incubated in the medium enriched with 150 µM of EAA (for 24 h);
- UVB + CBG: immediately after UVB radiation the cells were incubated in the medium enriched with 1 µM CBG (for 24 h);
- UVB + EAA + CBG: immediately after UVB radiation the cells were incubated in the medium enriched with 150 µM of EAA and 1 µM of CBG (for 24 h).

In the groups of the cells exposed to UVB radiation, the incubation with EAA and/or CBG took place immediately after the irradiation had been completed. The levels/activities of all the tested parameters were converted into mg of protein. The protein levels were determined through the Bradford test [30].

### 2.2. Methods

#### 2.2.1. Localization of EAA and CBG in Keratinocytes

The levels of 3-O-ethyl ascorbic acid and cannabigerol (CBG) were analysed by means of LC-MS/MS (QQQ) with an electrospray ionisation (ESI) source (LC-MS/MS 6460, Agilent, Kyoto, Japan) in the negative mode for 3-O-ethyl ascorbic acid and the positive mode for cannabigerol, employing multiple reaction monitoring (MRM). In short, CBG and EAA were isolated from the cytosol and membrane fraction of keratinocytes after protein precipitation. The samples were centrifuged for the purpose of the EAA determination, and the supernatant was analysed on a Zorbax SB-C18 (Agilent, Santa Clara, CA, USA) analytical column (4.6 mm × 150 mm; 3.5 µm particle size), following the method of Iliopoulos et al. [31] and monitoring *m*/*z* 203.1 → 85. The concentration of EAA was determined by means of a calibration curve range: 1–100 ng/mL (r^2^ − 0.9999). However, the lipophilic compound CBG was separated by SPE, and the actual separation was carried out on a Poroshell 120 EC-C18 analytical column (3.0 mm × 150 mm; 2.7 µm particle size), monitoring the following ions: 202.2 for CBG-d9 (internal standard) and 193.1 for CBG [32]. The obtained results are presented in terms of the percentage localisation of the tested compounds in the cytosol and membranes.

#### 2.2.2. Antioxidant Status of Keratinocytes

The total antioxidant status (TAS) was determined by means of ABTS (2,2′-azino-bis-3-ethylbenzothiazoline-6-sulfonic acid) [33]. ABTS radical solution was added to the keratinocyte lysates and incubated at 37 °C for 10 min. The absorbance was then measured by means of a microplate reader (EnSpire 2300 Multilable Reader; PerkinElmer, Waltham, MA, USA) at λ = 734 nm. The results obtained were expressed in terms of µmol of Trolox used as a standard (TEAC; Hoffman-LaRoche, Basel, Switzerland). The results were recalculated considering the protein content of each sample.

#### 2.2.3. Determination of the Activity of Enzyme-Metabolizing Phospholipids and Free PUFAs

The activity of cytosolic phospholipase A2 (PLA2–EC.3.1.1.4) in keratinocytes was assessed, by means of the colorimetrical analysis (λ = 405 nm), using a commercial assay kit (Cayman Chemical Company, Ann Arbor, MI, USA) [34]. The PLA2 activity is expressed in terms of nmol /min/mg of protein.

The activity of cyclooxygenases 1 and 2 (COX-1/2–EC.1.14.99.1) in keratinocytes was determined colorimetrically (λ = 590 nm) using a commercial assay kit (Cayman Chemical Company, Ann Arbor, MI, USA), monitoring the appearance of oxidised N,N,N0- and N0-tetramethyl-phenylenediamine (TMPD). The cyclooxygenase activity is expressed in terms of U/mg of protein.

The enzymatic activity of lipoxygenase (LOX-5) in keratinocytes was measured by means of the fluorescence method (Ex/Em 500/536 nm) using a commercial assay kit (Sigma-Aldrich, St. Louis, MO, USA). The LOX-5 activity in keratinocytes is expressed in terms of units per milligram of protein.

#### 2.2.4. Phospholipid and Free Fatty Acid Determination

The content of arachidonic acid (AA), eicosapentaenoic acid (EPA), and docosahexaenoic acid (DHA) (in both the phospholipid (PL) and free fatty acid (FFA) fractions) was qualified and quantified by means of gas chromatography combined with flame ionisation detection on Clarus 500 Gas Chromatograph (PerkinElmer, Waltham, MA, USA). The Varian CP-Sil88 capillary column was used in the analyses, with parameters such as length, inner diameter, and film thickness of 50 m, 0.25 mm, and 0.2 µm, respectively [35]. Briefly, fatty acids were extracted from the samples by means of the Folch method and then separated into the phospholipid fraction and free fatty acids using thin-layer chromatography (TLC), as described previously [36]. Fatty acid identification was based on a comparison of the retention times of the signals recorded for the analysed samples and standards, while quantification was performed by means of the internal standard (ISTD) method. Nonadecanoic acid (19:0) was used as the ISTD for FFA, while 1,2-dinonadecanoyl-sn-glycero-3-phosphocholine (19:0 PC) was used as the ISTD for the purpose of the PL analysis. The number of fatty acids were determined using calibration curves with a range of 1–100 µg/mL (r^2^ − 0.9994), 0.1–10 µg/mL (r^2^ − 0.9991), and 1–100 µg/mL (r^2^ − 0.9997) for AA, EPA, and DHA, respectively. The results were recalculated considering the protein content of each sample. The contents of AA (LLQ = 0.5 µg/mg protein), EPA (LLQ = 0.05 µg/mg protein), and DHA (LLQ = 0.5 µg/mg protein) are presented in terms of µg/mg of protein.

#### 2.2.5. Determination of the Level of Lipid Peroxidation Products

Changes in the levels of lipid peroxidation products in keratinocytes exposed to UVB radiation were assessed by measuring the levels of neuroprostanes (10-F4t-NeuroP; NPs) [37] and malonaldehyde (MDA) [38]. NPs were analysed using LC-MS/MS (QQQ) with an electrospray ionisation (ESI) source (LC-MS/MS 6460, Agilent, Kyoto, Japan) in the negative ion mode, employing MRM. In short, NPs were isolated from cell lysates by SPE after the alkaline hydrolysis step, which was described in greater detail in our previous works [39]. Separation was performed on an Eclipse Plus C18 column (2.1 × 100 mm; particle size 1.8 µm) in a gradient with solvents: (1) water (99.5%) and acetic acid (0.5%), (2) ACN (acetonitrile). Samples were analyzed by monitoring the following ions: 197.1 for 8-isoPGF2α-d_4_ (internal standard) and 153.0 for NPs. The level of NPs was determined on the basis of a calibration curve within the range of 5–300 pg/mL (r^2^ − 0.9991) and converted into mg of protein. The malondialdehyde content was measured by GC-MS/MS (7890A GC–7000 with a QQQ mass spectrometer, Agilent Technologies, Palo Alto, CA, USA). The MDA content was determined using GC-MS/MS (7890A GC-7000 with a triple quadrupole mass spectrometer, Agilent Technologies, Palo Alto, CA, USA). In order to evaluate the level of that lipid peroxidation product, the method proposed by Tsikas [38] was used, in which minor modifications had been made. The quantitative evaluation of MDA was based on its O-pentafluorobenzyl-oxime derivative content (*m*/*z* 204.0 and 178.0), while d3-4-HNE converted to an O-pentafluorobenzyl-oxime-trimethylsilane derivative served as the internal standard (*m*/*z* 245.0). A calibration curve for MDA within a range of 0.2 to 20 ng/mL (r^2^ − 0.9997) as well as the protein content of the samples were used for the purpose of calculations, which allowed the results to be presented in terms of ng/mg of protein.

#### 2.2.6. Determination of the Level of Eicosanoids

Ultra-performance liquid chromatography (UPLC) with a triple quadrupole mass spectrometer (QQQ) and with an ESI (electrospray ionization source), operating in the negative mode (Agilent Technologies, Palo Alto, CA, USA) were used for analysing pro- and anti-inflammatory eicosanoids: 15-hydroxyeicosatetraenoic acid (15-HETE), prostaglandin D2 (PGD2), prostaglandin E2 (PGE2), and 15-deoxy-Δ12,14-prostaglandin J2 (15-d-PGJ2) [40]. In short, the keratinocyte samples, upon application of the internal standards (15-HETE-d_8_, 15-d-PGJ2-d_4_, PGD2-d_4_—each with a concentration of 100 ng/mL)—were purified on SPE (Waters C18 Oasis, 3 mL, 200 mg). The method parameters: the column—Eclipse Plus C18 (2.1 × 100 mm; 1.8 µm); the mobile phase—the mixture of 0.1% CH3COOH in MilliQ water and ACN (in a gradient). The precursors to the product ion transitions were as follows: *m*/*z* 351.3 → 271.2 (PGE2 and PGD2), *m*/*z* 355.0 → 275.3 (PGD2-d_4_), *m*/*z* 315.2 → 271.2 (15-d-PGJ2), *m*/*z* 319.3→275.2 (15-d-PGJ2-d_4_), *m*/*z* 319.0 → 301.2 (15-HETE), and 327.0 → 226.2 (15-HETE-d_8_). The levels of PGE2, PGD2, 15d-PGJ2, and 15-HETE are expressed in terms of ng per milligram of protein.

#### 2.2.7. Estimation of the Endocannabinoid System

Ultra-performance liquid chromatography (UPLC) with a triple quadrupole mass spectrometer (QQQ) and with an ESI (electrospray ionization source), operating in the positive ion mode, were used for analysing the levels of endocannabinoids: arachidonoylethanolamine (anandamide, AEA), 2-arachidonoylglycerol (2-AG), and palmitoylethanolamide (PEA) [32]. In short, the keratinocyte samples, upon application of the internal standards (AEA-d_8_, 2-AG-d_8_, OEA-d_4_—each with a concentration of 100 ng/mL) were purified on SPE (Waters C18 Oasis, 3 mL, 200 mg). The method parameters: the column—Poroshell 120 EC C18 (3.0 mm × 150 mm; 2.7 µm); the mobile phase—the mixture of MilliQ water with formic acid (0.1%) and ACN (in a gradient). The precursors to the product ion transitions were as follows: *m*/*z* 348.3 → 62.15 (AEA), *m*/*z* 356.2 → 63.05 (AEA-d8), *m*/*z* 379.3 → 287.25 (2-AG), *m*/*z* 387.3 → 294.0 (2-AG-d8), *m*/*z* 300.3 → 62.0 (PEA), and *m*/*z* 330.20 → 66.15 (OEA-d_4_). The endocannabinoid levels in the keratinocytes are expressed in terms of pg per milligram of protein.

The spectrophotometric methods were used for determining the enzymatic activity of fatty acid amide hydrolase (FAAH-EC-3.5.1.99) and monoacylglycerol lipase (MAGL-EC 3.1.1.23). The FAAH determination method involves measuring the level of m-NA released from the FAAH substrate in lysed keratinocytes. The measurement was performed at a wavelength of 410 nm [41]. The FAAH activity in the keratinocytes are expressed in terms of nmol/min/mg protein. However, the MAGL method involves measuring the level of 5-thio-2-nitrobenzoic acid (TNB) released from DTNB (5,5-dithiobis (2-nitrobenzoic acid)). The measurement was performed at a wavelength of 412 nm [42]. The MAGL activity in the keratinocytes is expressed in terms of nmol/min/mg protein.

#### 2.2.8. Expression of Membrane Receptors

The enzyme-linked immunosorbent assay (ELISA) was used for assessing the expression of the membrane receptor proteins (TRPV1, CB1, CB2, PPARγ) [43]. In order to prepare the samples for the analysis purposes, the keratinocyte lysates were coated onto ELISA plates (Nunc Immuno Maxi Sorp, Thermo Scientific, Waltham, MA, USA) with the appropriate primary antibodies (all diluted 1:1000): TRPV1 (Sigma-Aldrich, St. Louis, MO, USA); CB1 and CB2 (Santa Cruz Biotechnology, Dallas, TX, USA) and PPARγ (Invitrogen, Thermo Fisher Scientific, Waltham, MA, USA) and then incubated at 4 °C overnight. After that step, the samples were washed with the solution of PBS and Tween 20 (0.1%) and incubated with the peroxidase blocking solution (30 min). Next, the secondary anti-rabbit/mouse EnVision+ Dual Link/HRP antibody (1:100) (Agilent Technologies, Santa Clara, CA, USA) was used and removed after 1 h of incubation. The samples were washed, and then the chromogen solution (0.1 mg/mL TMB with 0.012% H_2_O_2_) was added. The samples, with chromogen, were incubated for 40 min, and then the reaction was stopped by adding 2 mol/L H_2_SO_4_ solution. The assessment was conducted using a spectrophotometer by measuring the absorbance at 450 nm. The amounts of TRPV1, CB1, CB2, PPARγ in the keratinocytes were calculated by means of calibration curves within the following ranges: 0.1–10 ng/mL (CB1; Abcam, Cambridge, UK), 0.1–6 µg/mL (CB2; Abnova, Taipei, Taiwan), 1–150 pg/mL (PPARγ; Fine, Test Wuhan), 1–100 µg/mL (TRPV1; Lifespan Biosciences, Seattle, WA, USA), and the results were converted to mg of protein.

#### 2.2.9. Determination of Sialic Acid Level and Analysis of the Zeta Potential

In order to assess the total sialic acid content in the membranes, the modified Svennerholm’s resorcinol method was used [44]. The measurement was performed using a spectrophotometer at a wavelength of 630 nm. The N-acetylneuraminic acid solution was used as a standard, and the results were recalculated in consideration of the protein content of each sample.

The zeta potential of the cell membrane was determined in the keratinocytes suspended in 0.9% NaCl. The measurement was performed using the Zetasizer Nano ZS (Malvern Instruments, Malvern, UK), as described previously [45].

#### 2.2.10. Statistical Analysis

The statistical analysis was performed using GraphPad Prism 9 software. Comparisons between groups, where “n” for each group was 5, were made by one-way ANOVA followed by Tukey’s post-hoc test. Only *p* ≤ 0.05 was considered statistically significant, and the results are presented in terms of mean ± standard deviation (SD).

## 3. Results

### 3.1. EAA and CBG Cytosol and Membrane Localization

**Figure 1:**
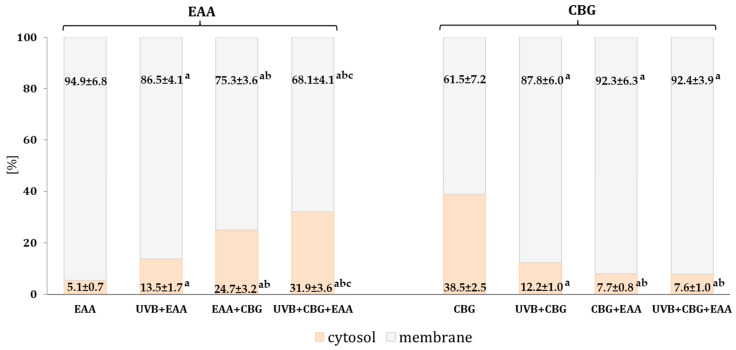
Percentage content of EAA or/and CBG in the cytosol and membrane fraction on the following groups of keratinocytes cultured for 24 h with EAA (150 µM) or/and CBG (1 µM), as well as cells exposed to UVB radiation (60 mJ/cm^2^) and then cultured for 24 h with EAA (150 µM) or/and CBG (1 µM). The mean ± SD values (n = 5) are presented with statistically significant differences: in cytosol or membrane a—vs. EAA (or CBG); b—vs. UVB + EAA (or UVB + CBG); c—vs. EAA + CBG (or CBG + EAA); *p* ≤ 0.05 formatting.

EAA and CBG added to the medium of keratinocytes (both control and UVB-irradiated) were primarily located in the membrane structures (Figure 1). However, in the keratinocytes exposed to UVB radiation, the amount of EAA in the membranes was reduced (by approximately 8%), while the level of CBG was increased (by approximately 26%). When both tested compounds were concurrently added to the medium, especially after the exposure to UVB, an increase in the level of EAA in the cytosol was observed: approximately 19% in the control cells and 26% in the irradiated cells, as compared to the group of control keratinocytes that received EAA alone. However, the level of CBG in the membranes of keratinocytes treated with the antioxidant duo (CBG + EAA) and in the UVB-irradiated cells treated with the same duo (CBG + EAA) was similar, accounting for approximately 5% of the level in the control cells treated only with CBG.

### 3.2. Total Antioxidant Status

**Figure 2:**
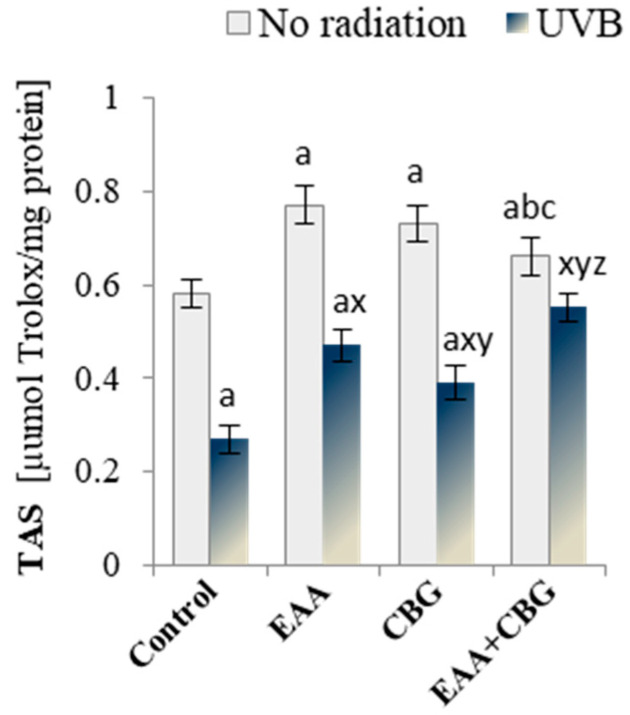
The effect of EAA or/and CBG on total antioxidant status (TAS) in the following groups of keratinocytes: control; cultured for 24 h with EAA (150 µM) or/and CBG (1 µM); as well as cells exposed to UVB radiation (60 mJ/cm^2^) and then cultured for 24 h with EAA (150 µM) or/and CBG (1 µM). The mean ± SD values (n = 5) are presented with statistically significant differences: a—vs. control group; b—vs. EAA group; c—vs. CBG group; x—vs. UVB radiation group; y—vs. UVB + EAA group; z–vs. UVB + CBG group; *p* ≤ 0.05.

The results of the conducted research indicate that exogenous compounds added to the keratinocyte medium, such as cannabigerol (CBG) and vitamin C derivative (EAA), support the endogenous antioxidant properties of cells. It is evident from the total antioxidant status (TAS) shown in Figure 2 that the greatest increase (32%) was observed with the addition of EAA alone. However, exposing keratinocytes to UVB radiation resulted in a 50% reduction in the TAS activity. As a result of the regenerative effect, EAA and CBG significantly increased the antioxidant capacity, by 74% and 44%, respectively, as compared to the values in the group exposed to UVB, and the concurrent use of both compounds (EAA + CBG) restored the TAS levels to those in the control group.

### 3.3. Composition and Functionality of the Keratinocytes Membrane

**Figure 3:**
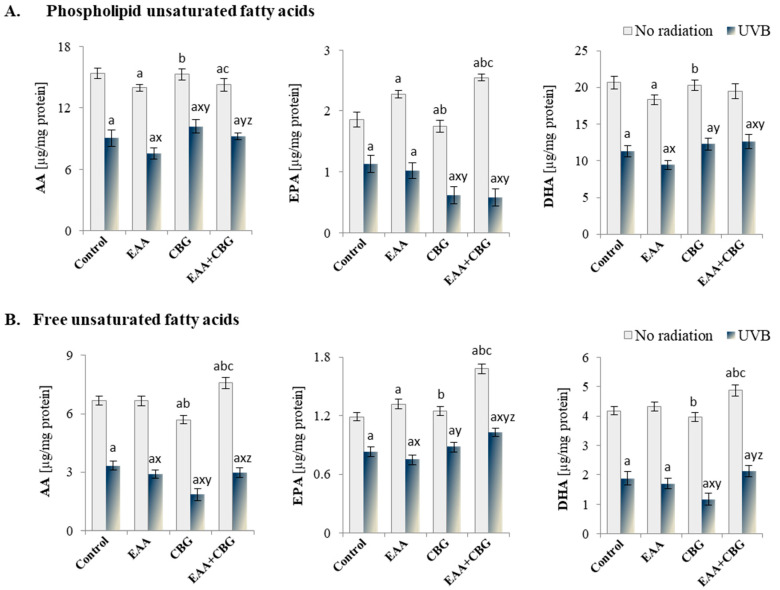
The effect of EAA or/and CBG on the level of (**A**) phospholipid (PL) and (**B**) free non-saturated fatty acids in the following groups of keratinocytes: control; cultured for 24 h with EAA (150 µM) or/and CBG (1 µM); as well as cells exposed to UVB radiation (60 mJ/cm^2^) and then cultured for 24 h with EAA (150 µM) or/and CBG (1 µM). The mean ± SD values (n = 5) are presented with statistically significant differences: a—vs. control group; b—vs. EAA group; c—vs. CBG group; x—vs. UVB radiation group; y—vs. UVB + EAA group; z—vs. UVB + CBG group; *p* ≤ 0.05.

Incubation of the control keratinocytes with the tested antioxidants also promoted changes in the composition and functionality of the cell membranes. It was particularly evident in the altered levels of phospholipid PUFAs, including the significant decrease in the levels of AA and DHA in the case of the EAA and EAA + CBG treatment as well as the increase in the level of EPA after the EAA, CBG, and EAA + CBG treatment, as compared to the control group (Figure 3A). In the case of free PUFA, only CBG reduced the level of AA, while the use of both antioxidants concurrently contributed to a significant increase in all of the three analysed PUFAs.

The exposure of keratinocytes to UVB radiation led to a significant reduction in the level of both free and analysed phospholipid PUFAs (Figure 3A,B), with the most noticeable changes in the level of phospholipid DHA (by approximately 45%) (Figure 3A) and free DHA (by 56%) (Figure 3B). Incubation of the cells exposed to UVB, given the EAA, CBG, and the EAA + CBG combination, caused different responses in the level of the acids tested. In the case of phospholipid PUFAs, the use of EAA decreased the levels of AA and DHA, while CBG, as compared to the UVB-irradiated cells, increased the level of AA and decreased the level of EPA. Moreover, the EAA + CBG combination reduced the level of EPA and increased the level of DHA. However, the level of free PUFAs changed as follows: EAA reduced the level of all the tested acids, CBG reduced the level of AA, and DHA significantly more than EAA and increased EPA, while the EAA + CBG combination promoted the reduction of the level of AA and increased the level of the remaining two acids.

**Figure 4:**
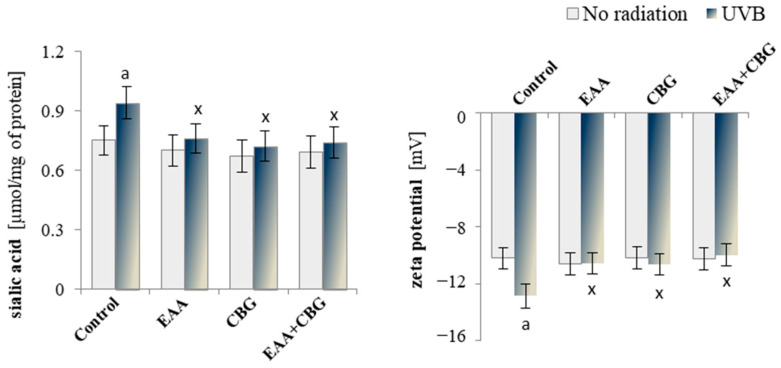
The effect of EAA or/and CBG on the level of sialic acid and zeta potential in the following groups of keratinocytes: control; cultured for 24 h with EAA (150 µM) or/and CBG (1 µM); as well as cells exposed to UVB radiation (60 mJ/cm^2^) and then cultured for 24 h with EAA (150 µM) or/and CBG (1 µM). The mean ± SD values (n = 5) are presented with statistically significant differences: a—vs. control group; x—vs. UVB radiation group; *p* ≤ 0.05.

The addition of EAA and/or CBG control keratinocytes to the medium did not cause statistically significant changes in the level of sialic acid, which is one of the main components of glycolipids and glycoproteins found in the cell membranes. However, UVB irradiation of the cells resulted in an increase in the level of sialic acid by approximately 25% as compared to the control cells (Figure 4), and the use of CBG, EAA, and EAA + CBG after UVB irradiation of keratinocytes significantly reduced the level of that acid (by approximately 30%, 24%, and 27%, respectively) as compared to the cells treated with UVB.

The changes in the level of sialic acid corresponded to the alterations in the zeta potential of the keratinocyte membrane (Figure 4). After UVB irradiation of the cells, the zeta potential was reduced by approximately 26% as compared to the control cells. However, the addition of the CBG, EAA, and the CBG + EAA combination to the medium after UVB irradiation of the cells increased the zeta potential by approximately 21%, 22%, and 29%, respectively, as compared to the cells irradiated only with UVB.

### 3.4. Phospholipid and Free PUFA Metabolism

A. ROS-dependent PUFA metabolism

**Figure 5:**
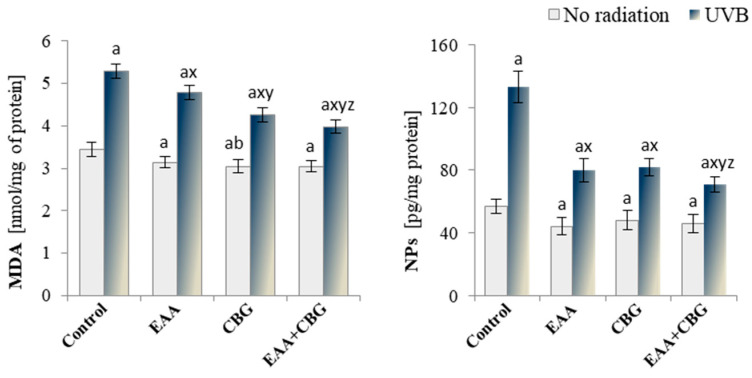
The effect of CBG or/and EAA on the level of lipid peroxidation products, malondialdehyde (MDA), and F4t-neuroprostane (NPs) in the following groups of keratinocytes: control; cultured for 24 h with EAA (150 µM) or/and CBG (1 µM); as well as cells exposed to UVB radiation (60 mJ/cm^2^) and then cultured for 24 h with EAA (150 µM) or/and CBG (1 µM). The mean ± SD values (n = 5) are presented with statistically significant differences: a—vs. control group; b—vs. EAA group; x—vs. UVB radiation group; y—vs. UVB + EAA group; z—vs. UVB + CBG group; *p* ≤ 0.05.

The increase in the level of TAS in the control keratinocytes treated with the compounds under consideration indicated their effective antioxidant activity, which was further demonstrated by the reduction in the lipid peroxidation products formed during both ROS-dependent peroxidation of phospholipids and free PUFAs (Figure 5). The exposure of keratinocytes to UVB radiation intensified the process of lipid peroxidation, evidenced by the increase in the level of small-molecule MDA (by approx. 54%) and neuroprostans (by approx. 133%). However, the addition of potential antioxidant compounds after the exposure to UVB, especially when used concurrently, resulted primarily in a significant decrease in the level of neuroprostanes.

B. Enzymes-dependent PUFA metabolism

**Figure 6:**
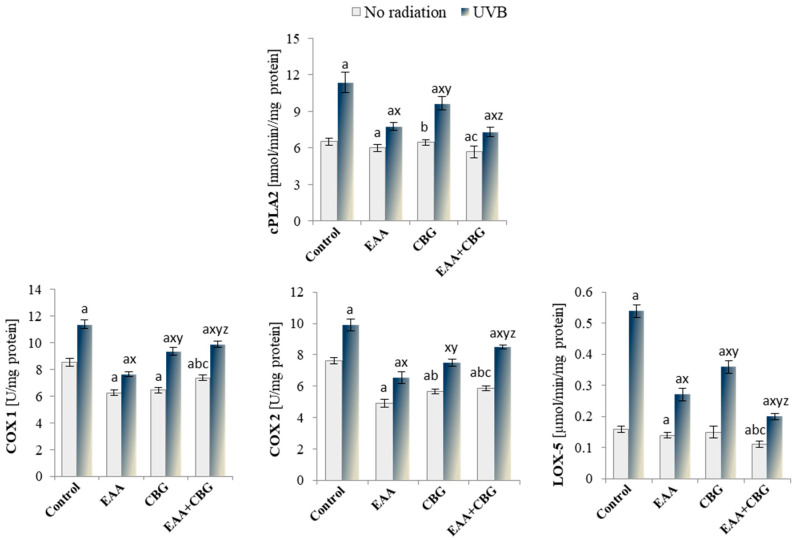
The activity of enzymes involved in the metabolism of eicosanoids cPLA2, COX1/2, and LOX-5 in the following groups of keratinocytes: control; cultured for 24 h with EAA (150 µM) or/and CBG (1 µM); as well as cells exposed to UVB radiation (60 mJ/cm^2^) and then cultured for 24 h with EAA (150 µM) or/and CBG (1 µM). The mean ± SD values (n = 5) are presented with statistically significant differences: a—vs. control group; b—vs. EAA group; c—vs. CBG group; x—vs. UVB radiation group; y—vs. UVB + EAA group; z—vs. UVB + CBG group; *p* ≤ 0.05.

EAA and CBG also had a significant impact on the enzymatic metabolism of phospholipids, which was observed as a statistically significant reduction in the activity of cytosolic phospholipase A2 (EAA and EAA + CBG) and enzymes that metabolise free PUFA, including COX1/2 and LOX-5 (mainly EAA + CBG) as compared to the control group (Figure 6). The exposure to UVB led to an increase in the activity of the tested enzymes, namely COX1/2, cPLA2, and LOX-5, by up to 237%. The used compounds significantly reversed those changes, favouring a reduction in the activity of the enzymes under consideration. EAA and the combination of compounds were most effective in reducing the activity of cPLA2 by approx. 35% and LOX-5 by approx. 63%. In the case of COX isoenzymes, EAA was the most effective, reducing their activity by approx. 30%.

**Figure 7:**
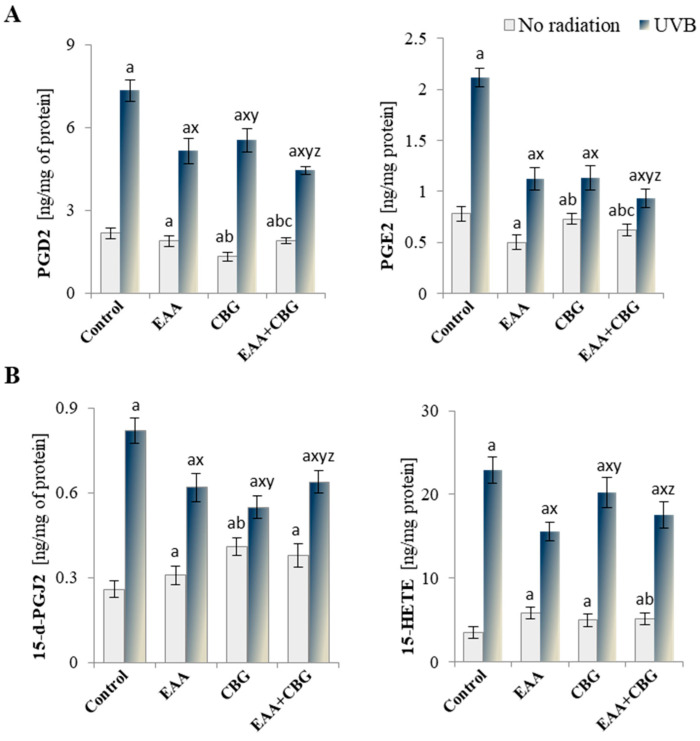
Level of (**A**) pro-inflammatory eicosanoids PGD2 and PGE2 and (**B**) anti-inflammatory eicosanoids 15-d-PGJ2 and 15-HETE in the following groups of keratinocytes: control; cultured for 24 h with EAA (150 µM) or/and CBG (1 µM); as well as cells exposed to UVB radiation (60 mJ/cm^2^) and then cultured for 24 h with EAA (150 µM) or/and CBG (1 µM). The mean ± SD values (n = 5) are presented with statistically significant differences: a—vs. control group; b—vs. EAA group; c—vs. CBG group; x—vs. UVB radiation group; y—vs. UVB + EAA group; z—vs. UVB + CBG group; *p* ≤ 0.05.

The changes in the activities of lipolytic enzymes resulted in the altered level of eicosanoids (Figure 7). The addition of EAA and CBG to the medium of the control keratinocytes increased the levels of anti-inflammatory eicosanoids (15-d-PGJ2 and 15-HETE) and decreased the level of pro-inflammatory prostaglandins (PGE2 and PGD2) when used both separately and in combination. UVB radiation significantly increased the levels of all the assessed eicosanoids, particularly 15-HETE, which rose by over 400%. That increase was partially counteracted by the antioxidant compounds, with the strength of their effect depending on the type of eicosanoid. In the case of pro-inflammatory eicosanoids (PGD2 and PGE2), the tested compounds caused a statistically significant reduction in their levels, and the changes after the use of EAA + CBG reached up to 30% and 35% for PGD2 and PGE2, respectively. Moreover, EAA and the combination of EAA + CBG reduced the levels of 15-d-PGJ2 and 15-HETE, which had been elevated after UVB irradiation of the cells.

**Figure 8:**
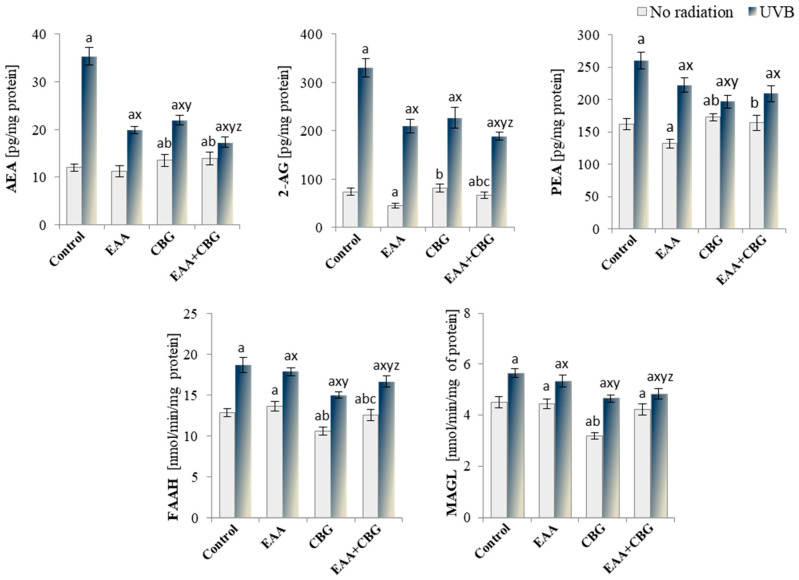
The changes in the endocannabinoid system measured as the level of endocannabinoids (AEA, 2-AG, PEA) and activity of their degrading enzymes (FAAH and MAGL) in the following groups of keratinocytes: control; cultured for 24 h with EAA (150 µM) or/and CBG (1 µM); as well as cells exposed to UVB radiation (60 mJ/cm^2^) and then cultured for 24 h with EAA (150 µM) or/and CBG (1 µM). The mean ± SD values (n = 5) are presented with statistically significant differences: a—vs. control group; b—vs. EAA group; c—vs. CBG group; x—vs. UVB radiation group; y—vs. UVB + EAA group; z—vs. UVB + CBG group; *p* ≤ 0.05.

Regardless of the above-discussed changes in the level of eicosanoids, the use of both antioxidants and the exposure of the cells to UVB radiation resulted in the changes in the levels of endocannabinoids generated from phospholipids through enzymatic reactions. In the control group, EAA and CBG used separately had different effects on the levels of the tested endocannabinoids, with EAA causing a decrease in the levels of 2AG and PEA, and CBG promoting an increase in the levels of AEA and PEA (Figure 8). UVB radiation intensified the metabolism of phospholipids, which resulted in a drastic increase in the level of all of the assessed endocannabinoids: PEA (by 60%), AEA (by 193%), and especially 2-AG by as much as 347%. The compounds had a regenerative effect on the keratinocytes and significantly reduced the endocannabinoid levels, with AEA and 2-AG decreasing by 51% and 43%, respectively, through the antioxidant combination. However, the PEA level was the lowest after the use of CBG and the EAA + CBG combination.

The level of endocannabinoids depends not only on the metabolic efficiency of phospholipids but also on the activity of enzymes that metabolise those compounds (FAAH and MAGL). Their activities were particularly effectively reduced by CBG, while UVB radiation significantly increased their activity (by approx. 45% FAAH and 26% MAGL). As a consequence, a reduction in the FAAH and MAGL activity was observed in the UVB-irradiated keratinocytes and those exposed to antioxidants, especially CBG and the EAA + CBG combination. However, the regenerative effect of both compounds used concurrently (EAA + CBG) was much more pronounced in the case of MAGL (Figure 8).

**Figure 9:**
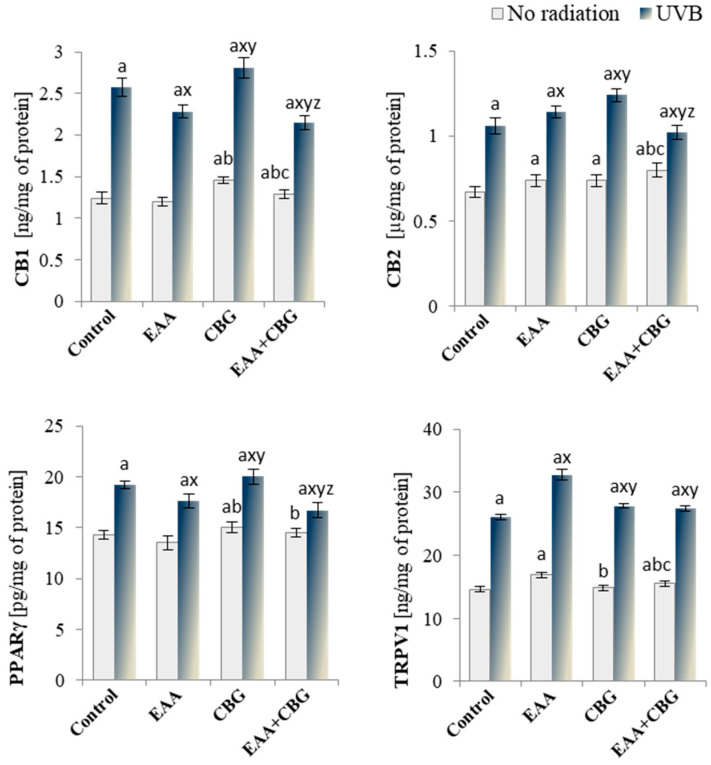
The level of membrane receptors activated by lipid mediators: CB1, CB2, PPARγ, and TRPV1 in the following groups of keratinocytes: control; cultured for 24 h with EAA (150 µM) or/and CBG (1 µM); as well as cells exposed to UVB radiation (60 mJ/cm^2^) and then cultured for 24 h with EAA (150 µM) or/and CBG (1 µM). The mean ± SD values (n = 5) are presented with statistically significant differences: a—vs. control group; b—vs. EAA group; c—vs. CBG group; x—vs. UVB radiation group; y—vs. UVB + EAA group; z—vs. UVB + CBG group; *p* ≤ 0.05.

Since both endocannabinoids and eicosanoids have metabolic effects, particularly in regulating the redox balance and inflammation by modulating the activation of G protein-coupled membrane receptors, the expression levels of key receptors were assessed. CBG caused an increase in the expression of the CB1/2 and PPARγ receptors as compared to the control group. However, EAA (alone and in combination with CBG) increased the expression of the CB2 and TRPV1 receptors, and the two-compound combination increased the expression of the CB1/2 and TRPV1 receptors (Figure 9). A significant increase in the expression of all the tested receptors was observed after exposing the cells to UVB radiation, with the greatest increase observed for CB1 (by 107%) and TRPV1 (by 78%). The use of EAA and CBG after UVB irradiation of the cells modified the expression of the above-mentioned receptors, with EAA increasing the expression of CB2 and TRPV1 and decreasing the expression of CB1 and PPARγ, while CBG led to an increase in the expression of all the tested receptors as compared to the group of the cells irradiated with UVB. However, the combination of both compounds reduced the expression of all the receptors except for TRPV1, the expression of which remained elevated.

## 4. Discussion

*Protective effect of EAA and CBG on the structure and functionality of membranes and metabolic processes of keratinocytes*

The biological effectiveness of protective and regenerative preparations in relation to skin cells arises from the ability of their ingredients to penetrate biological membranes as well as their metabolic efficiency at the level of both membranes and cytosol. Therefore, the use of preparations containing combinations of biologically active ingredients with diversified structures and, consequently, diverse physical properties, results in a different degree of penetration of cellular structures and, consequently, provides multidirectional possibilities of biological action [46]. That type of system uses a combination of phytocanabinoid—CBG, a compound with hydrophobic properties, and the hydrophilic-hydrophobic EAA. Consequently, EAA added to the keratinocyte medium in the control group promotes the intensification of the antioxidant efficiency of the keratinocytes, assessed in terms of the total antioxidant status (TAS), which may be related to the previously proven ability to directly scavenge free radicals, assessed on the basis of the reaction of EAA with the DPPH (2,2-Diphenyl-1-picrylhydrazylradical) [18]. Cannabigerol also has an antioxidant effect [24], which, as the results show, effectively increases TAS in the keratinocytes. Moreover, it has been shown that CBG also reduces the level of ROS in fibroblasts, which is attributed to its lipophilic nature and, thanks to that, its impact on the efficiency of the membrane NOX in the generation of superoxide anion radicals and hydrogen peroxide [25,47]. It is suggested that the main antioxidant effect is caused by the phenolic fragment of CBG, that enables the capture of cation radicals and their transformation into less active structures [48]. As a consequence, both compounds used in this study, used separately but especially in a two-component combination, applied to the medium of the control keratinocytes, have a protective and regenerative effect after UVB irradiation of the cells.

Consequently, taking into account the possible cellular localisation of EAA and CBG, the direction of action of those compounds pertains not only to the cytosol, but also, or perhaps primarily, to the membrane components of keratinocytes, including phospholipids. Those results show that the compounds used differentially modulate the levels of the tested PUFAs, with the strongest protective effect on phospholipid EPA and free AA, EPA, and DHA being observed when both compounds were used concurrently. However, the related literature indicates that EPA and DHA belonging to (n-3)PUFA may reduce the skin’s sensitivity to sunlight, reducing the tendency of human skin to erythema [49], which primarily confirms the protective effect of the EAA + CBG combination. Moreover, the related literature indicates that EPA is generally metabolised into anti-inflammatory eicosanoids, which provides the skin with effective anti-inflammatory and anti-cancer effects [50]. One of the main components of glycolipids and glycoproteins of the cell membrane is sialic acid, but the application of CBG and/or EAA to the medium of the control keratinocytes does not affect its level, suggesting that the compounds used do not disturb the sialylation of membranes, and thus the electrical properties, including the zeta potential of keratinocytes.

When used separately or in combination, the proposed compounds enhance the antioxidant effect of the cells and reduce the activity of phospholipase A2, thereby preventing the release of PUFA from phospholipids, as well as the oxidative oxidation of AA and DHA, which helps reduce the level of the PUFA peroxidation products (MDA and NPs). Those compounds are important signalling molecules in the functioning of cells. However, their generation reduces the continuity of biological membranes and also disrupts the basic metabolic processes of the cells [51]. It is even more important because MDA, due to its electrophilic nature, also has the ability to form complexes with nucleophilic centres of proteins, DNA, and lipids, which may modify their structures and functions [52].

The protective effect of the respective compounds and their two-component combination is also aimed at free PUFAs by reducing the activity of enzymes responsible for their metabolism (COX1/2 and LOX-5), which results in a reduction in the level of eicosanoids with pro-inflammatory properties (PGD2 and PGE2) and an increase in the level of eicosanoids with anti-inflammatory properties (15-d-PGJ2 and 15-HETE) [39]. It indicates not only the effectiveness of CBG and EAA in antioxidants but also anti-inflammatory activities. So far, it has been shown that CBG, by inhibiting the activity of phospholipase A2 (PLA2), interferes with the PGE2 synthesis pathway and leads to the inhibition of the activity of the constitutive isoform COX1 and COX2, thus demonstrating anti-inflammatory effects [53,54]. The above data suggest that the compounds used may regulate the intercorrelations between the redox balance and inflammation not only at the level of individual cells but also at the level of the entire skin.

As a result of the enzymatic metabolism of PUFAs, another group of lipid mediators, also important from the point of view of cellular metabolism, is created. Those are endocannabinoids, including those designated AEA, 2-AG, and PEA, that act as agonists of G protein-coupled receptors, including cannabinoid receptors (CB1/2), the activation of which promotes the regulation of the redox balance and inflammation [55]. Endocannabinoids are produced mainly in connection with the metabolic needs of the cell, while their metabolism involving FAAH and MAGL leads to the generation of free PUFA, mainly arachidonic acid, which is a precursor of many signalling molecules, including the previously mentioned eicosanoids [56]. Thus, the reduction in the FAAH and MAGL levels observed in the control group after the application of CBG inhibits the metabolism of 2-AG, AEA, and PEA. Moreover, it may be observed that CBG alone as well as in combination with EAA causes an increase in the expression of both CB1 and CB2 receptors, with the increase in the CB2 expression being greater and also affecting keratinocytes treated with EAA.

Taking into account the fact that the CB1/2 receptors are responsible for modifying the generation of ROS and TNFα, it may be expected that under the influence of the antioxidants, the activation of the CB2 receptors predominates, accompanied by a reduction in the level of ROS and TNFα, and consequently, the tendency to reduce oxidative stress and inflammation as well as modulation of the MAP kinase pathway [57]. It is also known that CBG may also indirectly affect the functioning of the endocannabinoid combination by inhibiting the uptake of anandamide, which leads to an increase in its level [58], which has been observed in this study. However, the increased level of AEA, which is also a TRPV1 agonist and is elevated by the antioxidants used, may intensify its pro-inflammatory effect. Reducing AEA levels, as has been shown in atopic dermatitis, helps decrease the resulting inflammation [59]. It is also known that CBG is a PPARγ agonist that, in the skin, controls the genetic regulation of genes involved in the inflammatory response, cell proliferation, and differentiation [60]. PPARγ induces a shift in the balance towards differentiation, which in turn leads to the normalisation of the final differentiation of the epidermal keratinocytes and a reduction in their proliferation [61]. Moreover, the anti-inflammatory activity of PPARγ may also be explained by inhibiting the expression of inflammatory markers, including NF-κB [62]. Moreover, the anti-inflammatory activity of PPARγ can also be explained by inhibiting the expression of inflammatory markers, including NF-κB [63]. Consequently, the tested compounds, by directly and indirectly regulating the cellular metabolism related to the redox balance and inflammation, ensure the proper functioning of the individual keratinocytes as well as the entire epidermis [64].

*Regenerative effect of EAA and CBG on the structure and functionality of membranes and metabolic processes of keratinocytes exposed to UVB radiation*

Although solar radiation is necessary for the proper functioning of the human body due to the stimulation of the biosynthesis of vitamin D and melanin and its effects on inhibiting the activity of microorganisms colonising the skin [65], excessive or chronic exposure of the skin to UVB radiation disturbs the cellular metabolism at the level of the redox balance and inflammation [66,67]. It results in intensified oxidative reactions [68], which usually account for a reduction of the antioxidant capacity [15] assessed in terms of TAS in this study. In such situations, the antioxidant compounds such as EAA and CBG may and do have a protective or regenerative function. So far, it has been shown that CBG effectively reduces the cellular ROS level increased by UVA/UVB radiation in the keratinocytes and fibroblasts [24,25] and reduces the proliferation of psoriatic keratinocytes stimulated by oxidative stress [69].

Pro-oxidant conditions resulting from UVB radiation [66] also constitute a direct cause of the phospholipid metabolism disorders dependent on ROS and lipolytic enzymes. The obtained results indicate that UVB radiation leads to a reduction in the level of phospholipid PUFAs, including AA, EPA, and DHA, which could suggest their increased release from phospholipids in the form of free acids [70]. However, it is not clearly confirmed by the reduced level of free PUFAs. In turn, phospholipid PUFAs, under oxidising conditions, undergo peroxidation [11], as evidenced by the increased level of cyclic neuroprostanes resulting from the oxidation of phospholipid DHA due to the significant amount of double bonds, in effect of the UVB exposure, which is most susceptible to the ROS-dependent metabolism [71]. At the same time, however, non-enzymatic peroxidation of free fatty acids occurs, producing low-molecular-weight aldehydes, including MDA [72]. As a consequence, the level of free fatty acids is significantly reduced after the exposure of the keratinocytes to UVB radiation. The use of EAA and CBG, especially the EAA + CBG combination, contributes to a significant reduction in the level of the lipid peroxidation products, which, however, does not result in a clear increase in the level of PUFA. The outcome indicates a varied response from phospholipid and free fatty acids and the type of PUFAs analysed (with a different number of double bonds). It has been previously shown that both tested compounds (EAA and CBG), when used in tandem, have a beneficial effect on keratinocytes exposed to UVA radiation. They effectively reduce the levels of another small molecular lipid peroxidation product, 4-HNE, and its adducts with proteins [73] while CBG significantly decreases the level of MDA in keratinocytes exposed to UVA radiation [25,74]. The related literature does not show the effect of EAA on the level of MDA in the keratinocytes exposed to UVB. However, it is known that the progenitor of EAA—ascorbate significantly protects skin cells against an increase in the level of MDA under oxidative stress, including that caused by UV radiation [75,76]. Therefore, taking into account the structural and functional similarity [18], it is plausible to suggest that the mechanism of metabolic action of EAA, due to its partially lipophilic nature, may be similar or even stronger in its action on lipophilic fatty acids.

Oxidative conditions accompanying UVB cell irradiation modify not only the levels of PUFAs but also other components of cell membranes, including sialic acid, which serves as a carrier of negative charge on the membrane surface. Under the influence of UVB radiation, the level of sialic acid in the keratinocytes increases, and the related literature confirms the relationship between the increase in the level of sialic acid and membrane sialylation intensified under the influence of UV radiation [64], which plays an important role in cell signalling and cell adhesion [45]. Moreover, an increase in the level of sialic acid contributes to a reduction in the zeta potential and changes in the physicochemical properties of the keratinocyte membrane, which is confirmed by the background and foreground knowledge [27,77]. As a consequence, the epidermal barrier is damaged, which causes the removal of intracellular water from keratinocytes through osmosis and cell shrinkage [78]. However, the use of EAA, CBG, as well as the EAA + CBG tandem reduces the harmful effects of UVB, which is crucial for the proper growth, migration, and regulation of the intracellular metabolism of the cells [72,78]. Moreover, since sialic acid is also considered a marker of inflammation in the cells, its lower level after the use of regenerative compounds also confirms the reduction of inflammation [79], which is a natural consequence of the exposure of the keratinocytes to UVB radiation [80].

Regardless of the modification of the structure and functionality of the membrane keratinocytes as a result of the UVB exposure, the creation of oxidative conditions also contributes to the increase in the activity of lipolytic enzymes responsible for the metabolism of phospholipids (PLA2) and PUFAs (especially LOX-5) with the generation of lipid mediators, including eicosanoids and endocannabinoids [81]. It additionally contributes to the reduced levels of AA, EPA, and DHA in the free PUFAs fraction. Both CBG and the CBG + EAA combination have the regenerative potential towards phospholipid fatty acids, especially AA and DHA, increasing their levels while reducing the cPLA2 activity. However, the level of EPA in the phospholipid fraction remains significantly reduced, while free EPA increases. EAA, on the other hand, reduces the activity of cPLA2 and also helps reduce the level of free AA and DHA. However, the related literature indicates that EPA and DHA, belonging to n-3 PUFAs, may reduce the skin’s sensitivity to sunlight, reducing the tendency to erythema caused by UV radiation [49]. Therefore, the increase in their level after the use of CBG and EAA + CBG indicates the beneficial effects of those compounds. Regardless of the modifications in lipolysis involving PLA2, UVB radiation promotes an increase in the activity of COX-1/2 and LOX-5, which are involved in the metabolism of free PUFAs, leading to the production of eicosanoids and consequently to an increase in the levels of all tested eicosanoids (PGE2, PGD2, and 15-d-PGJ2) produced under the influence of COXs, as well as 15-HETE generated with the participation of LOX-5 [39,82].

It has already been shown that an increase in the level of EPA and DHA in the keratinocytes correlates with a decrease in the level of PGE2 [39], which may also be observed in the presented results. Moreover, it is known that EPA competes with arachidonic acid in the metabolic processes of keratinocytes, potentially reducing the production of their metabolites, including PGE2 [83]. Regardless of the metabolic changes analysed above, PUFAs, COXs, and LOXs also generate metabolic pathways producing leukotrienes B4 (LTB4), which indirectly, through the increase in the expression of the BLT2 receptor, increases the level of ROS, thereby leading to an increased oxidative stress and activation of apoptosis [84,85].

However, the application of EAA, CBG, and their two-component combination to the medium of the keratinocytes exposed to UVB reduces the level of all the determined eicosanoids, which may be the result of the reduced activity of enzymes involved in their biosynthesis by the antioxidants used. CBG, especially when used with EAA, reduces the level of PGE2, which is accompanied by an increase in the levels of both EPA and DHA. The recent data indicate that the reduction of the PGE2 levels is accompanied by an increase in the collagen production, thus inhibiting the ageing process, which is evident after the use of another phytocannabinoid, cannabidiol [86]. Moreover, in Alzheimer’s disease, CBG has been proven to have the ability to reduce the level of AA, which is an important mediator of inflammation in the skin [53,87], especially after the exposure to UVB radiation when the arachidonic acid inflammatory pathway is activated [88]. CBG has also been shown to reduce the IL-1β-induced PGE2 production in gingival fibroblasts [89]. However, it has been found that the much more thoroughly tested phytocannabinoid—CBD, by inhibiting the activity of phospholipase A2 (PLA2), interferes with the prostaglandin E2 (PGE2) synthesis pathway, thus demonstrating anti-inflammatory effects, consequently improving the condition of the skin [90]. Thus, it is plausible to suggest that CBG and its combination with EAA may have an equally beneficial effect on the skin regeneration. However, it is known that vitamin C, as a precursor of EAA, leads to a reduction in the level of PGE2 in the human neuroblastoma cell line (SK-N-SH) [91]. Moreover, it has been found that, regardless of their inflammation-modulating effect, eicosanoids, especially PGD2 and its metabolite 15-d-PGJ2, may be involved in the regulation of the inflammatory signals generated by PPARγ [92]. The increased levels of both compounds after having exposed the keratinocytes to UVB were significantly reduced after the use of EAA and CBG, especially when used in combination. In the same way, those compounds reduced the LOX-5 activity of the keratinocytes induced by UVB radiation, consequently leading to a reduction in 15-HETE, an anti-inflammatory lipoxin [39].

Another group of lipid mediators that play an important regulatory role in both the redox activities and pro-inflammatory signalling of the keratinocytes is the group of endocannabinoids. Their metabolic action is achieved through the activation of G-protein-related receptors, mainly CB1 and CB2 [93]. In the keratinocytes irradiated with UVB, the levels of AEA and 2-AG are increased more than three times, and the level of PEA is significantly increased. Therefore, despite an approximately 20% increase in the activity of enzymes responsible for the metabolism of AEA and 2-AG (FAAH and MAGL), those changes may significantly influence the modification of the cellular metabolism. The increased activity of FAAH and MAGL as well as 2-AG, AEA, and PEA has previously been observed after the exposure of the skin cells (keratinocytes and fibroblasts) to UVA/B radiation [39,89,94]. EAA and CBG, used both separately and in combination, significantly reduce the effects of radiation by significantly reducing the level of endocannabinoids, especially AEA and 2AG, which results in a reduction in the expression of CB1/2, TRPV1, and PPARγ membrane receptors [95]. The increase in the expression of the CB1 receptor in particular, observed under the influence of UVB, confirms the intensification of the pro-oxidant-pro-inflammatory conditions in the cells, as the increased expression of this receptor has been associated with the increased production of reactive oxygen species (ROS) and the release of tumour necrosis factor alpha (TNFα), which activates the NFκB transcription factor. However, the increased expression of the CB2 receptor promotes the reduction of oxidative stress and pro-inflammatory signalling [55,96].

The reduction in the CB1 receptor expression observed after the use of EAA and the EAA + CBG combination indicates a partial reduction of both oxidative stress and pro-inflammatory signalling in the keratinocytes after the exposure to UVB radiation, which may be considered an exceptionally effective effect of EAA, as the use of CBG alone tends to further intensify the expression of the assessed receptors. It is surprising given the existing literature on CBD, which indicates a reduction in the expression of cannabinoid receptors and their derivatives [82]. Moreover, CBG is known to be active in the Ca^2+^ and Na^+^ channels [53], exhibiting partial agonism towards the peroxisome proliferator-activated receptors (PPARγ) [54], which may directly increase the gene expression with antioxidant and anti-inflammatory properties [97]. However, EAA and, to a lesser extent, CBG, and the combination of the compounds, intensify the expression of the TRPV1 receptor, already increased by UVB, which, after activation by endogenous and exogenous inflammatory mediators, is responsible for the release of neuropeptides and the neurogenic inflammatory response in the cells, as a result of which it is closely related to the ageing skin and the development of chronic inflammatory skin diseases (e.g., psoriasis, atopic dermatitis, or rosacea) [59]. Therefore, the observed additional increase in the expression of that receptor, especially after the application of EAA to the medium of keratinocytes exposed to UVB rays, may suggest further development of inflammation in keratinocytes. However, it is known that CBG is one of the few compounds, next to CBD, that stimulates TRPV1 receptors and anaesthetises them to block the transmission of pain signals [96,98]. Additionally, the increase in the TRPV1 levels may be associated with the increase in the Nrf2 levels, which has been observed after the application of CBG to the keratinocytes previously exposed to UVA radiation [25], which indirectly confirms its anti-inflammatory effect.

## 5. Conclusions

The obtained results indicate that the use of ethyl derivative of vitamin C (EAA) and CBG, especially in combination, enhances the antioxidant capacity of the keratinocytes, which promotes the protection/regeneration of membrane phospholipids. The antioxidants used significantly reduce the metabolism of phospholipids, which is intensified by UVB radiation, and consequently reduce the levels of lipid mediators (eicosanoids and endocannabinoids) involved in the modulation of both oxidative stress and inflammation. However, in order to assess the global metabolic response of the cells to the action of the proposed compounds, proteomic and lipidomic assessment of the keratinocytes will also be needed. The current results only indicate the possibility of using those compounds to counteract the effects of excessive sun exposure and to treat skin diseases. However, taking into account that those results are preliminary and pertain only to the response of the keratinocytes in vitro, in order to fully verify the assessment of the response of the analysed compounds, in vivo studies will also be necessary, including the response of the keratinocytes to the application of the tested compounds to the skin of animals, but above all, patients in clinical trials.

## Data Availability

All data generated or analyzed during this study are included in this article.

## Supplementary Materials

The following supporting information can be downloaded at: https://www.mdpi.com/article/10.3390/antiox13111285/s1.
