# Supplementary material for: 3-O-Ethyl Ascorbic Acid and Cannabigerol in Modulating the Phospholipid Metabolism of Keratinocytes
Source: Antioxidants (Basel). 2024 Oct 24;13(11):1285. doi: 10.3390/antiox13111285 (PMC11591156; doi:10.3390/antiox13111285)
Supplement: Supplementary file 1 [file antioxidants-13-01285-s001.zip › antioxidants-3227125-supplementary.pdf]

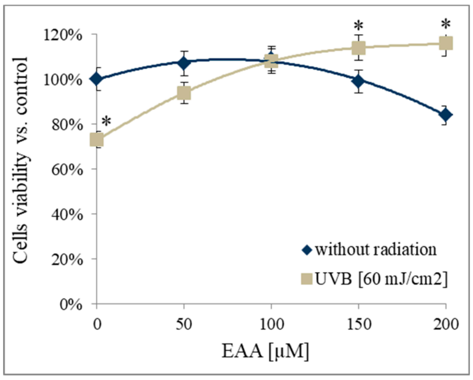

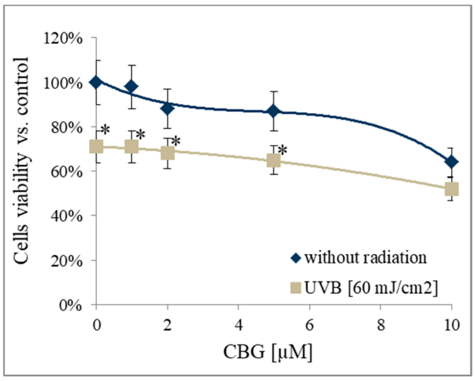

Figure S1. The viability of keratinocytes exposed to UVB radiation [60mJ/cm^2] and treated with 3-O-ethyl ascorbic acid [EAA, 50-200 µM] or cannabigerol [CBG, 1 - 10 µM]. Analyzes were performed according to the MTT assay protocol. The mean±SD values (n=3) are presented with statistically significant differences:
*statistically significant differences vs. non-irradiated group, p<0.05.

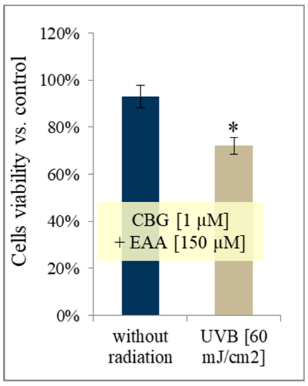

Figure S2. The viability of keratinocytes exposed to UVB radiation [60mJ/cm^2] and treated with both compounds at the selected concentration after together used [150 µM EAA and 1 µM CBG]. Analyzes were performed according to the MTT assay protocol. The mean±SD values (n=3) are presented with statistically significant differences:
*statistically significant differences vs. non-irradiated group, p<0.05.
